# Release of Infectious Hepatitis C Virus from Huh7 Cells Occurs via a *trans*-Golgi Network-to-Endosome Pathway Independent of Very-Low-Density Lipoprotein Secretion

**DOI:** 10.1128/JVI.00826-16

**Published:** 2016-07-27

**Authors:** Jamel Mankouri, Cheryl Walter, Hazel Stewart, Matthew Bentham, Wei Sun Park, Won Do Heo, Mitsunori Fukuda, Stephen Griffin, Mark Harris

**Affiliations:** a School of Molecular and Cellular Biology, Faculty of Biological Sciences, University of Leeds, Leeds, United Kingdom; b Leeds Institute of Cancer and Pathology, Faculty of Medicine and Health, St. James' University Hospital, Beckett St., Leeds, United Kingdom; c Department of Biological Sciences, Korea Advanced Institute of Science and Technology, Daejeon, Republic of Korea; d Graduate School of Life Sciences, Tohoku University, Sendai, Miyagi, Japan; Washington University School of Medicine

## Abstract

The release of infectious hepatitis C virus (HCV) particles from infected cells remains poorly characterized. We previously demonstrated that virus release is dependent on the endosomal sorting complex required for transport (ESCRT). Here, we show a critical role of *trans*-Golgi network (TGN)-endosome trafficking during the assembly, but principally the secretion, of infectious virus. This was demonstrated by both small interfering RNA (siRNA)-mediated silencing of TGN-associated adaptor proteins and a panel of dominant negative (DN) Rab GTPases involved in TGN-endosome trafficking steps. Importantly, interfering with factors critical for HCV release did not have a concomitant effect on secretion of triglycerides, ApoB, or ApoE, indicating that particles are likely released from Huh7 cells via pathways distinct from that of very-low-density lipoprotein (VLDL). Finally, we show that HCV NS2 perturbs TGN architecture, redistributing TGN membranes to closely associate with HCV core protein residing on lipid droplets. These findings support the notion that HCV hijacks TGN-endosome trafficking to facilitate particle assembly and release. Moreover, although essential for assembly and infectivity, the trafficking of mature virions is seemingly independent of host lipoproteins.

**IMPORTANCE** The mechanisms by which infectious hepatitis C virus particles are assembled and released from the cell are poorly understood. We show that the virus subverts host cell trafficking pathways to effect the release of virus particles and disrupts the structure of the Golgi apparatus, a key cellular organelle involved in secretion. In addition, we demonstrate that the mechanisms used by the virus to exit the cell are distinct from those used by the cell to release lipoproteins, suggesting that the virus effects a unique modification to cellular trafficking pathways.

## INTRODUCTION

Hepatitis C virus (HCV) is a major cause of chronic hepatitis, which can progress to cirrhosis and hepatocellular carcinoma. The recent development of direct-acting antivirals (DAA) (1), exemplified by the recently FDA-approved polymerase inhibitor sofosbuvir, promises much improved treatment of HCV. However, high drug costs, coupled with concerns over resistance, require a deeper understanding of HCV biology in order to identify novel antiviral targets.

HCV is an enveloped virus with a 9.6-kb positive-sense single-stranded RNA genome, encoding a 3,000-amino-acid polyprotein precursor which is cleaved by host and viral proteases into the structural proteins core, envelope proteins E1 and E2, and p7 and the nonstructural (NS) proteins NS2, NS3, NS4A, NS4B, NS5A, and NS5B. Core protein, the principle constituent of the viral capsid, is targeted to lipid droplets (LDs) (2–4), implying a role for these lipid storage organelles during HCV assembly. The E1 and E2 glycoproteins form a heterodimeric complex in the viral envelope. NS5A and viral RNA colocalize with core protein in proximity to LDs (5), and an interaction between NS5A and core is critical for the production of infectious virions (6). Furthermore, NS2 interacts with p7, E2, NS3, and NS5A, coordinating these proteins to bring them into proximity with LDs during virion assembly (7–10). A late-acting, postassembly role for NS2 during virion release has also been described previously (8, 11, 12).

The cellular pathways involved in virion assembly and release remain poorly understood. Assembly of infectious intracellular HCV virions occurs in close proximity to LDs and is dependent on factors required during very-low-density lipoprotein (VLDL) assembly, including DGAT1 (13), apolipoprotein B-100 (ApoB) (14), and ApoE (15). A role for VLDL pathways in the subsequent release of HCV particles has therefore been proposed although not formerly demonstrated (reviewed in reference 16). More recently, we, along with others, demonstrated that HCV release is dependent on components of the endosomal sorting complex required for transport (ESCRT), suggesting that HCV release is dependent on endosomal compartments (17, 18). In addition, early endosome proteins such as Rab5 have been shown to be required for virus genome replication, suggesting that HCV is dependent on this compartment at multiple stages in the virus life cycle (19, 20).

Here, we further dissect the endosomal routes of HCV egress. We show that *trans*-Golgi network (TGN)-associated adaptors play a pivotal role during both HCV assembly and release. Furthermore, a TGN-secretory endosomal pathway, defined by dominant negative (DN) Rab-GTPase mutants, mediates the release of infectious virions but not that of triglycerides (TGs) or ApoE, the principle components of VLDL. We also show that the TGN redistribution observed within HCV-infected cells is driven by NS2 but only when it is expressed in the context of a functional HCV replicase. Thus, our data support the idea that release of infectious HCV occurs via a TGN-endosome secretory pathway that is distinct from that of VLDL within infected Huh7 cells.

## MATERIALS AND METHODS

### Cell culture.

Huh7 cells were cultured as described previously (21). Subgenomic replicon-harboring cell lines were generated and maintained as described previously (22).

### DNA constructs and transfection.

Wild-type (WT) and dominant negative (DN) Rabs were expressed using a pEGFP-C1 or pECFP-C1 (where EGFP is enhanced green fluorescent protein and ECFP is enhanced cyan fluorescent protein) vector and have been described previously (23). A total of 2 × 10^5^ Huh7 cells in one well of a six-well plate were transfected using polyethylenimine (PEI) (Polysciences, Inc.) according to the manufacturer's instructions. To quantify absolute numbers of transfected cells, plates were visualized with an IncuCyte Zoom system (Essen Bioscience) at 24 h posttransfection, and the number of green objects per well was calculated. Filter parameters were set to exclude objects with <200-μm^2^ and >3,000-μm^2^ areas, thereby excluding cellular debris. Background threshold was set to a minimum of 5.0 green calibrated units (GCU) and was increased where necessary until an untransfected control well had a score of zero.

### Production of infectious HCV.

A total of 10 μg of *in vitro*-transcribed (Promega) JFH-1, Jc1 (24), or J6/JFH-1luc (a luciferase-expressing J6/JFH-1 chimeric virus) (25) transcripts was electroporated into Huh7 cells, which were washed and resuspended in diethyl pyrocarbonate (DEPC)-treated phosphate-buffered saline (PBS) (8 × 10^6^ cells; 400 μl). Electroporation was carried out using a Bio-Rad Gene Pulser II at 975 μF and 270 V in a 4-mm cuvette. Cells were plated into T175 flasks in 12 ml of medium, which was harvested/replenished every 24 h to generate pooled virus stocks.

### siRNAs and transfection.

Validated Silencer Select small interfering RNAs (siRNAs) (Life Technologies) were used to silence AP1MI, AP2MI, GGA1, GGA2, and GGA3. Huh7 cells were seeded (1 × 10^5^ cells) into each well of a six-well plate and transfected with 150 pmol of each siRNA, including a scrambled RNA control, using Lipofectamine RNAiMax (Life Technologies) according to the manufacturer's instructions. At 48 h posttransfection, the supernatant and cell lysate were collected for analysis, and/or cells were infected with virus as described below.

### Quantification of effects on HCV particle production and release.

Following transfection (see above), cells were infected at a multiplicity of infection (MOI) of 0.2 focus-forming units (FFU)/cell for 24 h in complete Dulbecco's modified Eagle's medium (DMEM). Following two washes in PBS, released virus was collected in serum-free medium for 24 h. Clarified culture supernatant infectivity was determined by focus-forming assay. This was also performed for intracellular infectivity following resuspension of cells in 50 μl of PBS, five repetitive freeze-thaw cycles, and clarification at 2,800 × *g* in a microcentrifuge for 5 min. Data are expressed as means and standard errors. Statistical significance was determined using a paired Student *t* test for comparison of two sets of data or a one-way analysis of variance (ANOVA) with a Bonferroni test when data for more than two Rab constructs were compared against the GFP-alone controls. A *P* value of <0.05 was deemed significant.

### Quantification of secreted triglyceride/ApoE.

Triglyceride content of heat-inactivated (65°C, 15 min) phenol red and serum-free culture supernatants was assessed using a colorimetric lipase-based assay that hydrolyzes triglycerides to free glycerol, according to the manufacturer's instructions (Sigma). For Western blot analysis of ApoE levels, supernatants were precipitated with methanol at 4°C overnight; thereafter, precipitated lipoproteins were pelleted at 10,000 × *g* for 10 min and resuspended in 1× Laemmli buffer for Western blot analysis. Cells were lysed in Glasgow lysis buffer (GLB; 10 mM PIPES [piperazine-*N*,*N*′-bis(2-ethanesulfonic acid]-KOH, pH 7.2, 120 mM KCl, 30 mM NaCl, 5 mM MgCl_2_, 1% [vol/vol] Triton X-100, and 10% [vol/vol] glycerol) plus protease inhibitors (Roche Complete) and phosphatase inhibitors (2 mM Na_3_VO_4_, 5 mM NaF, 5 mM Na_4_P_2_O_7_), and 10 μg of total protein was analyzed by Western blotting. Secreted ApoB and ApoE were also detected and quantified with commercial enzyme-linked immunosorbent assay (ELISA) kits (AlerCHEK). Briefly, supernatants were diluted 1:10 with diluent (ApoB ELISA) or used undiluted (ApoE ELISA) and allowed to adhere to the plate for 45 min. After plates were thoroughly washed, horseradish peroxidase (HRP)-conjugated goat anti-human ApoB/ApoE secondary antibody was added and allowed to incubate for 45 min. Wells were washed and incubated with 3,3′,5,5′-tetramethylbenzidine (TMB)-peroxide solution for 15 min before the reaction was terminated with sulfuric acid. Absorbance was read at 450 nm, and sample values were extrapolated from a positive-control standard curve.

### Replication assays.

Rab-GFP/CFP-expressing or siRNA-treated cells were infected with J6/JFH-1luc at an MOI of 0.2 FFU/cell as described above prior to lysis in 400 μl of passive lysis buffer (Promega). Renilla luciferase activity was measured using dual-luciferase Stop and Glo reagent (Promega) using a luminometer (EG&G Berthold). All assays were performed in triplicate, and each experiment was repeated a minimum of three times. All data are expressed as means and standard errors.

### Western blotting.

Infected or transfected cells were lysed in GLB as described above, and 10 μg of protein was resolved by SDS-PAGE. Proteins were transferred onto polyvinylidene difluoride (PVDF) membrane (Millipore) using a Bio-Rad semidry transfer apparatus and then probed with mouse anti-ApoE antibody (Sigma or Abcam), polyclonal rabbit anti-core protein (kind gift from John McLauchlan, Centre for Virus Research, Glasgow, Scotland), rabbit polyclonal anti-AP1M1, -AP2MI, -GGA1, -GGA2, or -GGA3 (Abcam), mouse monoclonal anti-EGFP, or mouse anti-glyceraldehyde 3-phosphate dehydrogenase (GAPDH) (GeneTex). Washed membranes were incubated with HRP-conjugated donkey anti-sheep, donkey anti-rabbit, or goat anti-mouse secondary antibody (Sigma) and visualized using an in-house enhanced chemiluminescence system.

### Immunofluorescence microscopy.

Cells grown on glass coverslips were fixed for 10 min with 3% (vol/vol) paraformaldehyde (PFA) in PBS at room temperature or in ice-cold methanol, followed by permeabilization in ice-cold methanol-acetone for 10 min. Cells were washed with PBS and blocked in PBS–1% bovine serum albumin (BSA) for 30 min prior to incubation with primary antibodies for 1 h in PBS–1% BSA with polyclonal rabbit anti-core protein, sheep polyclonal anti-TGN46 (kind gift from Sreenivasan Ponnambalam, University of Leeds) (0.5 μg/ml), mouse monoclonal anti-AP1 (Sigma), rabbit polyclonal anti-NS5A serum (kind gift from Ralf Bartenschlager, University of Heidelberg), rabbit polyclonal anti-AP2M1 (Abcam) or mouse monoclonal AP33 anti-E2 antibody (provided by Genentech). Washed cells were then labeled using an appropriate Alexa Fluor 488/594/647-conjugated secondary antibody (Invitrogen). Cells were washed and mounted onto microscope slides using Citifluor (Agar Scientific) and then viewed on a Zeiss 510-META laser scanning confocal microscope under an oil immersion 63× objective lens (numerical aperture, 1.40). Alexa Fluor 488 dye (494-nm excitation; 519-nm emission) was excited using an argon laser fitted with 488-nm filters, Alexa Fluor 594 dye (550-nm excitation; 570-nm emission) was excited using a helium-neon laser fitted with 543-nm filters, and Alexa Fluor 647 dye (650-nm excitation; 670-nm emission) was excited using a helium-neon laser fitted with 633-nm filters. Images displayed are representative and are displayed as single optical sections.

## RESULTS

### HCV particle assembly and release require specific TGN adaptors.

Given the emerging evidence implicating a role for the endosomal trafficking machinery during HCV egress, we employed siRNA targeting of key TGN-endosomal trafficking proteins, including the μ1 subunits of the TGN-resident clathrin adaptor complex AP1 and the endocytic adaptor AP2 (termed AP1M1 and AP2M1), as well as the Golgi-localized gamma adaptin ear-containing, ARF-binding (GGA) proteins GGA1, GGA2, and GGA3, to assess their roles in virus assembly/egress. Huh7 cells transfected with an siRNA were subsequently infected either with JFH-1 to assess infectious particle production or with a luciferase-expressing J6/JFH-1 chimeric virus (J6/JFH-1luc) to measure genome replication.

**FIG 1:**
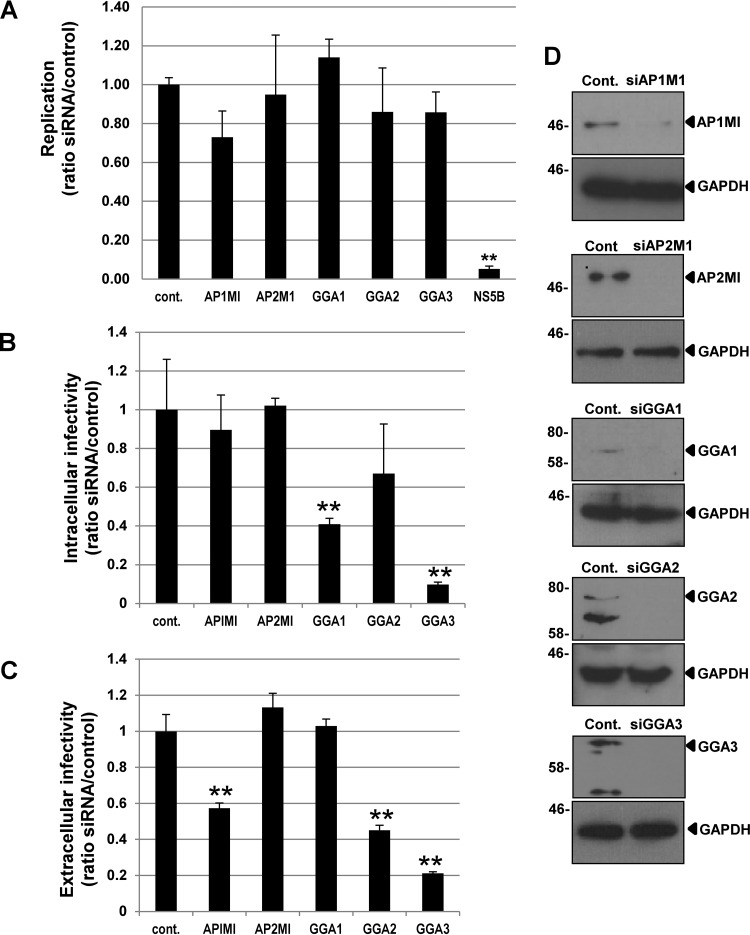
Effects of siRNA knockdown of Golgi apparatus-endosome trafficking proteins on HCV genome replication and virus production. Huh7 cells were transfected with siRNAs for each of the five targets or with a scrambled RNA control and incubated for 48 h, followed by infection with JFH-1 at an MOI of 0.2 for 48 h. (A) For the assessment of HCV genome replication, cells were infected with J6/JFH-1luc at an MOI of 0.2 for 48 h prior to measurement of luciferase activity. Extracellular (B) and intracellular (C) infectivity were then assessed by focus-forming assay. All data are expressed as the ratio of absolute values obtained in the presence of the scrambled RNA control to those of the siRNA target sample and are presented as means plus standard errors. **, *P* < 0.05. (D) At 72 h posttransfection, cells transfected with either a control scrambled siRNA (Cont.) or targeted siRNAs, as indicated, were lysed and probed for each target protein and GAPDH, as an internal control, by Western blot analysis.

As expected, an siRNA targeted to the NS5B coding region of the virus genome efficiently blocked genome replication whereas silencing of individual TGN trafficking proteins had no significant effects (Fig. 1A). In contrast, the importance of TGN-endosomal trafficking to both virion assembly and egress was evident as the siRNA mediated disruption of intracellular and secreted infectivity. Depletion of AP1M1 had no effect on intracellular infectivity (Fig. 1B) but inhibited release (44% ± 2.5% reduction in extracellular infectivity) (Fig. 1C). Similar data have recently been reported with the silencing of the TGN-endosome adaptor AP1 gamma subunit (AP1G1) in JFH-1-infected hepatocytes (26). This same trend in infectivity was observed when GGA2 was silenced (64% ± 3.5% reduction in extracellular infectivity) (Fig. 1C), suggesting that both this and AP1M1 strongly influence the delivery of assembled virions into the secretory pathway. In contrast, a lack of GGA3 resulted in significant reductions in both intracellular (87% ± 4% reduction) (Fig. 1B) and extracellular (78% ± 3% reduction) (Fig. 1C) infectivity, suggesting that virus assembly had been disrupted. Intriguingly, silencing of GGA1 yielded an unusual phenotype whereby intracellular infectivity was reproducibly diminished (60% ± 4.5% reduction) whereas that in the secreted compartment was unaltered (Fig. 1B and C). We further consider the implications of this result in the discussion. In contrast to previous studies (27) and further supporting a TGN-mediated pathway of virus egress, knockdown of AP2M1 (an endocytic adaptor) had no effect on the virus life cycle. Efficient silencing of individual proteins was confirmed in Huh7 cells by Western blotting (Fig. 1D). This is in agreement with another more recent study in which knockdown of the AP2M1 was shown to have no effect on extracellular HCV RNA release (26). Together, these data strongly support the critical importance of the TGN and its associated adaptors during HCV particle production.

### Secretory, but not endocytic, Rab-GTPases control the release of HCV particles.

**FIG 2:**
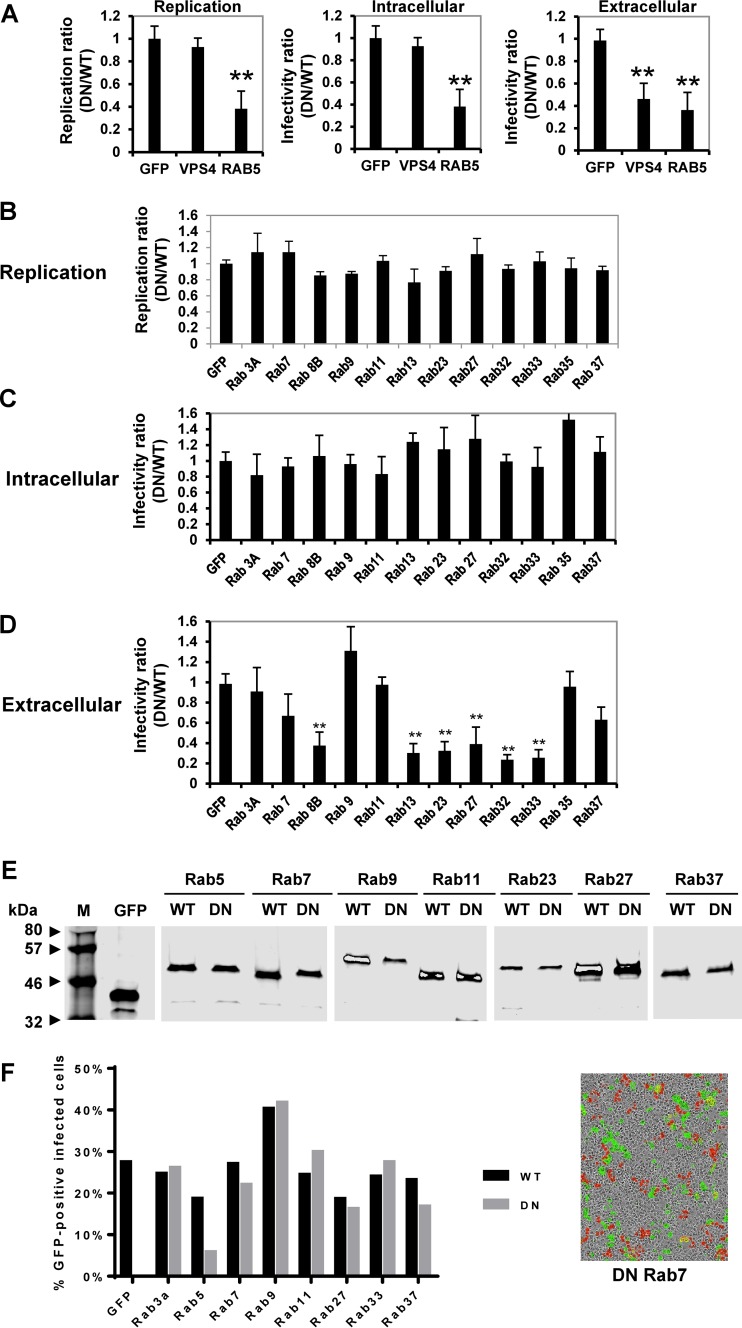
HCV release is controlled by secretory, but not endocytic, Rab-GTPases. Huh7 cells were transfected with plasmids expressing either wild-type or DN versions of the indicated proteins for 24 h, followed by infection with JFH-1 at an MOI of 0.2 for 24 h. Infectivity was determined by focus-forming assay. For the assessment of HCV genome replication, cells were infected with J6/JFH-1luc at an MOI of 0.2 for 48 h prior to measurement of luciferase activity. (A) Validation of the assay using previously characterized controls: VPS4, which inhibits assembly, and Rab5, which inhibits genome replication. (B to D) Results of a replication assay and intracellular and extracellular infectivity assays are shown, as indicated. All assays were performed in triplicate, and each experiment was repeated a minimum of three times. All data are expressed as the ratio of absolute values obtained in the presence of the wild-type (WT) to those of the DN Rab construct (DN/WT) and are presented as means and standard errors. **, *P* < 0.05. (E) To analyze Rab protein expression, the indicated cell lysates were subjected to Western blotting for EGFP. Lane M, molecular mass marker. (F) To determine the efficiency of the transfection/infection procedure, the numbers of infected cells (NS5A positive) that were also GFP positive were manually counted, and results are displayed as a percentage of the total number of infected cells. A representative image of DN Rab7 transfected cells is shown for illustrative purposes.

Given the clear importance of TGN-endosomal adaptors during particle production, we reasoned that virions likely follow a defined endosomal pathway during egress. To map this pathway, we exploited the large family of Rab GTPases (>60 members), individual members of which govern specific membrane trafficking steps. Huh7 cells were transfected with a panel of plasmids encoding wild-type or DN Rab proteins as GFP/CFP fusions prior to infection with JFH-1 or J6/JFH-1luc. Effects on intracellular/secreted infectivity or replication (assayed by luciferase activity) were calculated by dividing the value obtained in the presence of the DN by that obtained for the corresponding wild-type Rab; values of less than 1 thus represented a defect mediated by the DN Rab. We first verified that different stages of the HCV life cycle could be discriminated using DN mutants of cellular cofactors: Rab5, which interacts with NS4B and is required for HCV RNA replication (20), and the AAA-family ATPase VPS4, which mediates budding/release of assembled HCV particles via recycling of ESCRT-III (17, 18). Reassuringly, DN Rab5 reduced HCV replication (61% ± 10% inhibition) with concomitant effects on total infectivity (62% ± 9% inhibition), whereas DN VPS4 specifically reduced secreted infectivity (64% ± 8% inhibition) with no effect on replication or intracellular infectivity (Fig. 2A).

We therefore extended our analysis to include Rab proteins governing either endocytic/recycling (Rab7, -9, -11, and -35) or secretory/exocytic (Rab3a, -8b, -13, -23, -27, -32, -33, and -37) pathways (Fig. 2). No effects upon replication (and by inference, virus entry) (Fig. 2B) or on the production of intracellular particles (Fig. 2C) were apparent, yet several DN secretory/exocytic Rabs significantly inhibited the release of infectious HCV; DN Rab8b, -13, -23, -27, -32, and -33 proteins caused ≥60% decrease in extracellular titers (Fig. 2D). Endocytic/recycling Rab proteins lacked an appreciable role during particle assembly or egress. Comparable expression was confirmed for a subset of the Rab proteins (those expressed as GFP fusions) by Western blotting (Fig. 2E); while overall levels expectedly varied to some extent between Rabs, variation within pairs of WT/DN constructs was similar. Thus, where DN Rabs affected particle release (e.g., Rab23 and -27), it is highly unlikely that this resulted from increased expression relative to the WT counterpart. Furthermore, we confirmed that transfected cells were not somehow rendered refractory to infection by quantifying the proportion of NS5A-expressing cells that were also GFP positive using the IncuCyte Zoom system (28). This revealed similar levels of infection in all cases except where the DN endocytic Rab5 was expressed, which might be expected, given its role during replication, as discussed above. Moreover, given that only a subset of infected cells expressed dominant negative proteins, it is likely that our population-based infectivity measures in fact underestimate the effects on release at the level of individual transfected cells. We therefore conclude that the trafficking/release of assembled, infectious HCV particles involves transfer through the TGN into a defined secretory endosomal pathway within Huh7 cells.

### Perturbation of the TGN/endosomal axis of HCV assembly/release does not affect VLDL secretion.

Current models assume that HCV co-opts the VLDL secretory machinery to effect the release of infectious virions, and this assumption is based upon the essential role for factors involved in VLDL biogenesis during particle assembly and the modulation of VLDL synthesis and secretion by infected cells (29). However, direct evidence for this secretory pathway is lacking, and recent studies show that clathrin-mediated stages of virion egress appear distinct to ApoE secretion; knockdown of either clathrin or its adaptor AP1 impaired virus secretion but had no effect on extracellular ApoB or ApoE (26). Hence, in order to reconcile our observed TGN/endosomal dependence of secretion with these observations, it was necessary to formally investigate whether knockdown/disruption of TGN adaptors or Rabs led to disruption of VLDL secretion. To test this, experiments were repeated with cells grown under serum-free conditions to permit direct measurement of released ApoE, ApoB, and triglyceride, which comprise the major elements of the VLDL-like particles generated by Huh7 cells (30).

**FIG 3:**
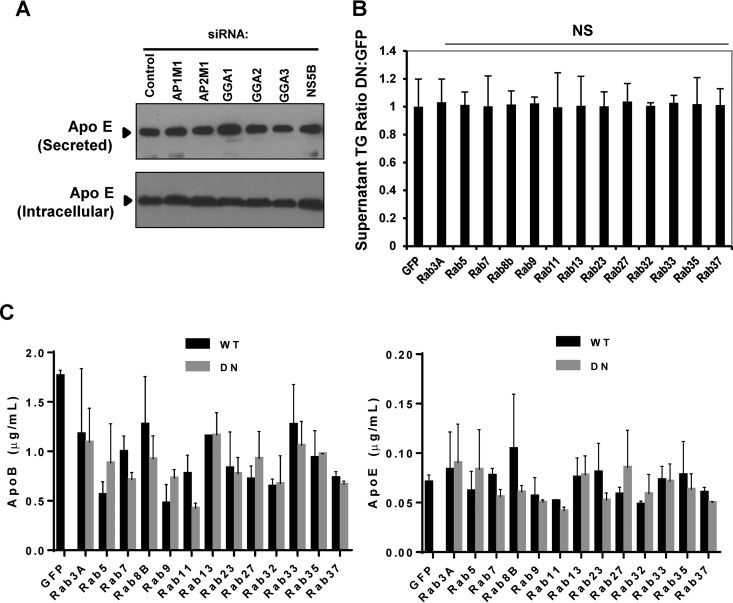
Inhibition of TGN-endosomal trafficking proteins or secretory Rabs does not affect VLDL secretion. (A) To assess ApoE trafficking and secretion in siRNA-transfected cells, ApoE was methanol precipitated from the supernatants and assessed by Western blot analysis. Cell lysates were also probed for intracellular ApoE by Western blot analysis. (B) Secreted triglyceride levels were assessed in the culture supernatants from the HCV-infected cells from the experiment shown in Fig. 2 using a colorimetric lipase-based assay that hydrolyzes triglycerides to free glycerol. NS, not significant. (C) To assess secretion of ApoE and ApoB from WT or DN plasmid-expressing cells, culture supernatants were analyzed by ELISA.

We first examined whether ApoE release was affected by knockdown of factors that reduced the accumulation of intracellular HCV infectivity (GGA1 and -3), as well as that of the release-specific AP1M1 and GGA2. Levels of both intracellular and secreted ApoE were unaffected, with the exception of a modest increase in secreted ApoE in the case of GGA1 knockdown (Fig. 3A). This was expected as GGA1 has been shown to specifically associate with endocytosis of the ApoE receptor, LR11/SorLA (31). We then quantified the effects of perturbing Rab protein function on the secretion of the major constituent of VLDL, triglyceride (TG). Again, no effect on TG release was observed, regardless of whether DN Rab proteins affected HCV particle production or not (Fig. 3B). Last, we directly quantified the secretion of both ApoE and ApoB using ELISAs; as shown in Fig. 3C, levels of both ApoB and ApoE secretion were inherently variable, yet this variation did not correlate with observed effects upon secreted infectivity (compare Fig. 2D and 3C).

**FIG 4:**
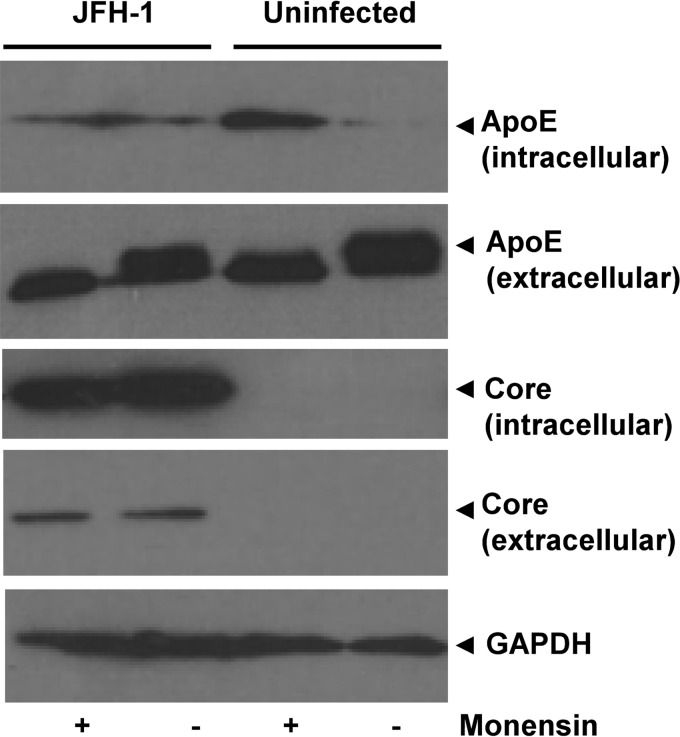
Core protein secretion can be separated from apolipoprotein secretion. JFH-1-infected or uninfected cells were treated with monensin (2 μM) for 18 h. Lysates or precipitated supernatants were analyzed by Western blotting for the indicated proteins.

Next, we took the converse approach of inhibiting VLDL secretion using monensin and asking whether this adversely affected the secretion of HCV proteins into the extracellular milieu (Fig. 4). Monensin effectively reduced extracellular ApoE levels and led to the concomitant intracellular accumulation of ApoE, confirming a block to secretion. Under these conditions there was no effect on either intracellular or extracellular levels of core protein. This is consistent with a recent publication demonstrating inhibitory effects of monensin on HCV entry but a lack of effect on assembly or release of infectious virus (32). Thus, while VLDL components clearly play a vital role during virion morphogenesis, the TGN/endosomal pathways specifically concerned with HCV trafficking/release appear separate from those governing VLDL secretion.

### TGN morphology and trafficking are altered during HCV infection.

**FIG 5:**
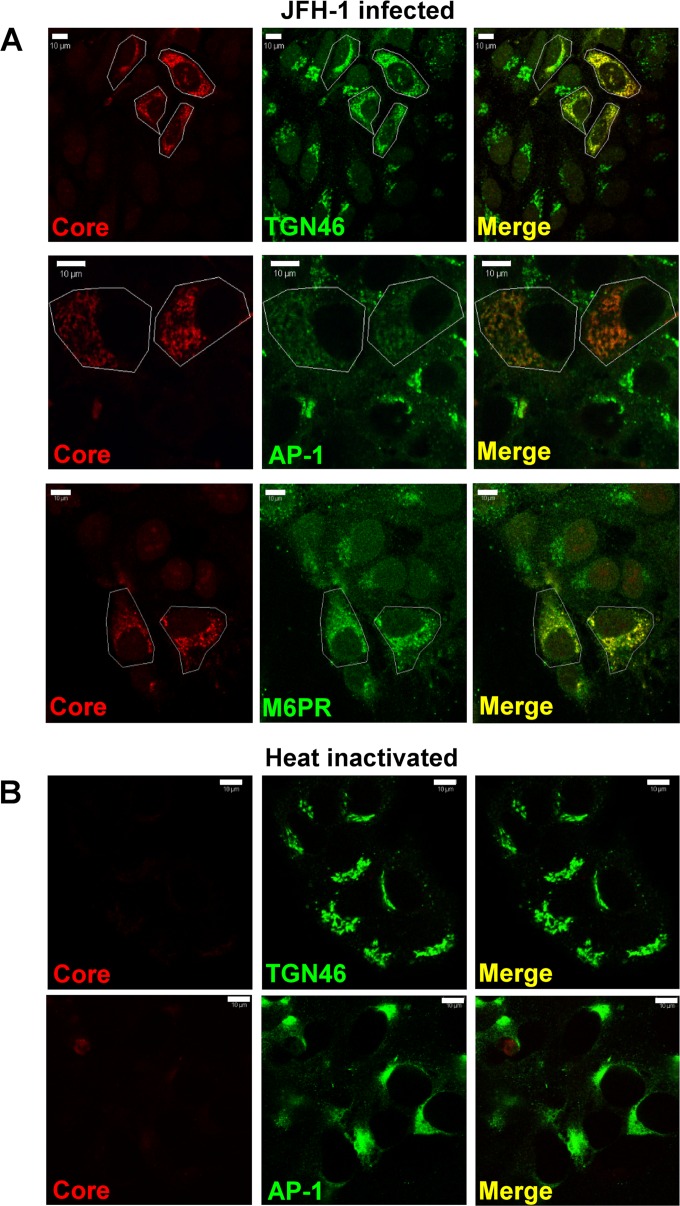
HCV infection alters TGN morphology. (A) Huh7 cells were infected with JFH-1, fixed, and permeabilized prior to staining with a rabbit anti-core antibody and Alexa Fluor 594-conjugated anti-mouse secondary antibody. Endogenous TGN46, AP1, and M6PR were detected using either a sheep polyclonal anti-TGN46 or mouse monoclonal anti-AP1 and M6PR antibodies with Alexa Fluor 488-conjugated anti-sheep or anti-mouse secondary antibodies. (B) Virus stocks were heat inactivated at 65°C for 30 min prior to infection of Huh7 cells, and TGN and AP1 staining were assessed as described for panel A. Representative wide-field images are shown. Scale bar, 10 μm.

Given the role of the TGN during HCV assembly/release, we next assessed changes to TGN membrane architecture during HCV infection. Consistent with previous studies (33), we found that the TGN markers TGN46 and AP1 exhibited a dispersed distribution in JFH-1-infected cells compared to that in surrounding uninfected cells, which retained a characteristic perinuclear Golgi “stack” (Fig. 5A). TGN redistribution was further confirmed by staining infected cells for the TGN-endosome shuttling protein mannose 6-phosphate receptor (MPR) (34, 35). MPR was again more widely distributed within infected cells than in adjacent naive cells. TGN redistribution was dependent on productive virus entry and replication as infection with heat-inactivated virus prevented alteration of TGN46 or AP1 distribution (Fig. 5B). These data support the observation that HCV disperses the TGN and alters the normal trafficking profile of TGN-resident proteins.

### TGN membranes are contiguous with putative HCV assembly sites in infected cells.

**FIG 6:**
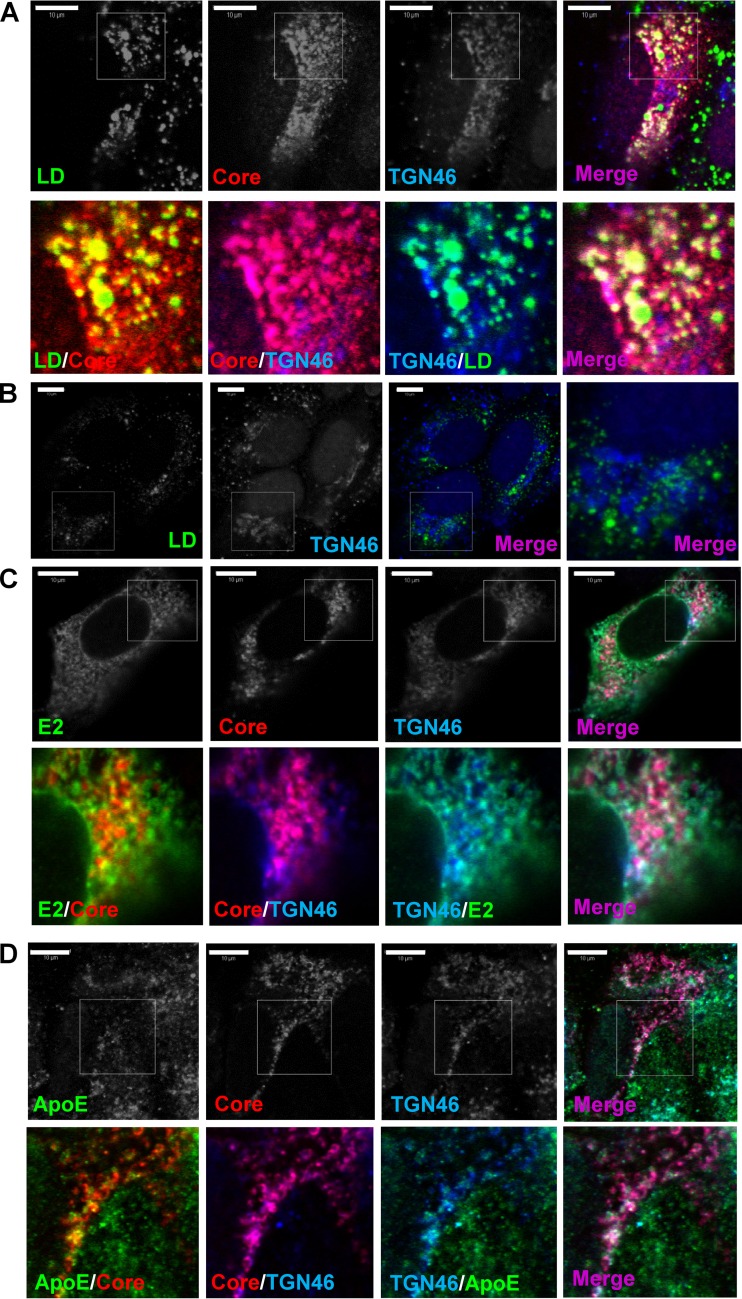
Redistribution of the TGN to the sites of HCV assembly. Huh7 cells were infected with JFH-1 (A, C, and D) or mock infected (B), fixed, and permeabilized prior to staining with anti-core protein and anti-TGN46 (as described in the legend to Fig. 5), together with BODIPY for detection of lipid droplets (LDs) (A and B), mouse anti-E2 antibody (AP33) (C), or mouse monoclonal anti-ApoE antibody (D) and appropriate secondary antibodies. Representative confocal images are shown. Scale bar, 10 μm.

We noted that in JFH-1-infected cells, the pattern of TGN staining was reminiscent of that previously reported for LDs. To investigate this further, we costained JFH-1-infected cells with antibodies to TGN46 and core protein, together with the lipid reactive dye BODIPY (dipyrromethene boron difluoride) (Fig. 6A). All three components showed contiguous distributions such that the redistributed TGN compartments were juxtaposed with both LDs and core protein within infected cells (Fig. 6A), but not in uninfected cells (Fig. 6B), in a pattern reminiscent of the coating of LDs reported for core protein and NS5A (5, 36, 37).

We considered that the redistribution of the TGN results in juxtaposition of this compartment with HCV assembly sites (2, 5). While E2 and the core protein were only partially contiguous with each other, noticeable clustering of both proteins occurred at areas positive for TGN46 (Fig. 6C). Such clustering also occurred for ApoE (Fig. 6D) and most likely represents the sites of virus assembly. We therefore consider that TGN membrane redistribution within close proximity to LDs occurs during virus infection, potentially linking regions of virion assembly with the secretory pathways required for HCV egress.

### NS2-mediated TGN alterations within infected cells require a functional HCV replicase.

**FIG 7:**
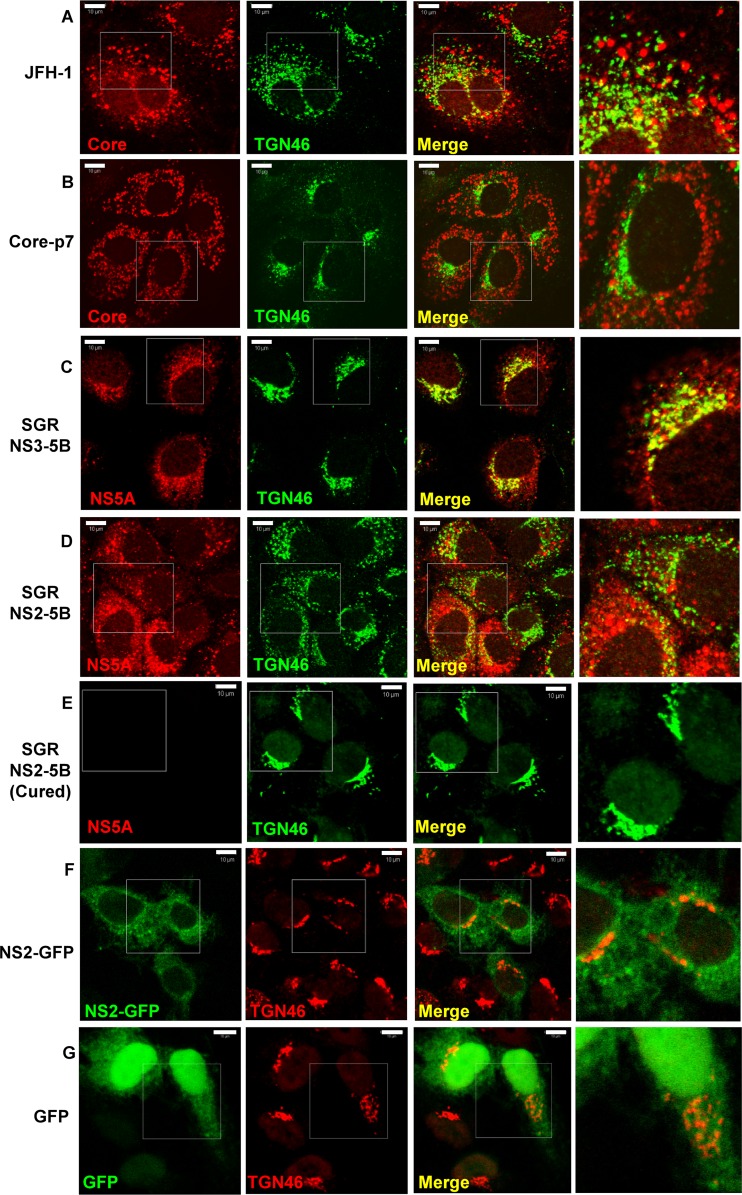
NS2 is necessary, but not sufficient, to mediate TGN redistribution. Huh7 cells were infected with JFH-1 (A) or transiently transfected with a plasmid expressing core-p7 (B), fixed, and permeabilized prior to staining for core protein and anti-TGN46, as described in the legend to Fig. 5. Huh7 cells stably harboring a JFH-1-derived NS3-5B subgenomic replicon (SGR) (C) or a modified version also containing NS2 (D) were fixed and permeabilized prior to staining for NS5A with rabbit anti-NS5A polyclonal antiserum and Alexa Fluor 594-conjugated anti-rabbit secondary antibody and TGN46 as described in the legend to Fig. 5. (E) NS2-5B subgenomic replicon-harboring cells were cured by treatment with interferon (100 units/ml for 3 weeks) and stained as described for panel C. Huh7 cells were transfected with a plasmid expressing an NS2-GFP fusion (F) or pEGFP-N1 (G), fixed, and permeabilized prior to staining for TGN46 with sheep polyclonal anti-TGN46 and Alexa Fluor 594-conjugated anti-sheep secondary antibody. Representative confocal images are shown. Scale bar, 10 μm.

**FIG 8:**
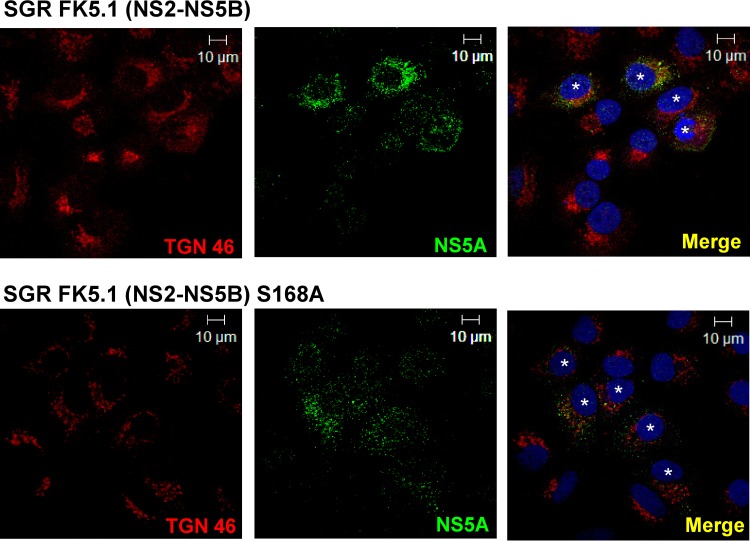
NS2 serine 168 is not required for TGN redistribution. Huh7 cells stably harboring G418-resistant genotype 1b FK5.1 (culture-adapted) subgenomic replicons (SGRs) were fixed and permeabilized prior to costaining with a rabbit anti-NS5A antibody and sheep anti-TGN46 antibody, followed by labeling with Alexa Fluor 488-conjugated anti-rabbit and Alexa Fluor 594-conjugated anti-sheep secondary antibodies. Subgenomic replicon-positive cells are highlighted with a white asterisk in the nucleus. Representative confocal images are shown. Scale bar, 10 μm.

Given the role of NS2 in coordinating NS and structural proteins during virion assembly, we hypothesized that NS2 might be responsible for the TGN rearrangements. To test this, we expressed either the structural proteins (core protein-p7 expression), the minimal HCV replicase (NS3 to NS5B [NS3-5B] replicon), or the complete nonstructural protein repertoire (NS2-5B replicon) (38) within Huh7 cells. Strikingly, compared to results in JFH-1-infected cells (Fig. 7A), the expression of core-p7 alone displayed no discernible effects on TGN architecture, and a mutually exclusive staining pattern for core protein and TGN46 was observed (Fig. 7B). Similarly, the TGN was not redistributed within cells expressing an NS3-5B replicon although some overlap of NS5A (a surrogate marker for replication complexes) with TGN46 was apparent (Fig. 7C). However, cells expressing an NS2-5B replicon recapitulated the TGN rearrangements observed during full virus infection (Fig. 7D). TGN rearrangements required continuous HCV gene expression and were not a clonal selection artifact as a normal TGN appearance was restored within NS2-5B replicon cells cured by interferon treatment (Fig. 7E). Furthermore, the context of NS2 expression within the viral replicase appeared critical as NS2 fused to GFP expressed alone did not induce redistribution of the TGN (Fig. 7F), as was the case for control cells transfected with GFP alone (Fig. 8G). Finally, we assessed whether the functionally conserved NS2 Ser168 might play a role during this process; Ser168 is a target for phosphorylation by casein kinase II (39) and strongly influences both infectious particle production (8, 40) and NS5A interactions (38). Replicons containing the S168A mutation still induced TGN rearrangements (Fig. 8), indicating that this process requires additional NS2 functional determinants. Furthermore, these replicons were based on the genotype 1b culture-adapted FK5.1 sequence (22), indicating that the ability to induce TGN rearrangements is not restricted to the genotype 2a JFH-1 isolate.

## DISCUSSION

The starting point for this study was the critical importance of TGN-endosomal adaptors during HCV particle production. Silencing of the μ1 subunit of AP1, an adaptor involved in TGN-endosome trafficking, inhibited virus egress but did not affect virus replication/assembly. Encouragingly, this same result has been achieved with the silencing of the γ1 subunit of AP1 (26). Our study also agrees with the findings of Benedicto and colleagues (26) in that no reduction of infectivity was observed when the μ1 subunit of AP2 (an endocytic adaptor) was silenced, making TGN-endosome trafficking the key route of HCV JFH-1 egress (Fig. 1). However, a previous study (27) showed that the production of infectious J6/JFH-1 chimeric virus is reduced following AP2M1 knockdown. The discrepancy between these studies may reside in the differential core protein localizations in J6/JFH-1 and JFH-1, which in turn are dictated by p7 and NS2 and suggest that these viruses may co-opt related, yet distinct, pathways during egress (41).

GGA silencing yielded further insights into the TGN-endosomal transport of HCV particles. Silencing of GGA1 reduced the levels of intracellular virus but did not influence virus replication or extracellular titers. These data imply that GGA1 may play a role in stabilizing intracellular virus. In this regard it is intriguing that GGA1 (but not GGA2 or -3) plays a role in the endocytosis of the ApoE receptor LR11/SorLA, a member of the LDL receptor family (31). By reducing endocytosis of ApoE, GGA1 silencing could either inhibit the process of HCV assembly indirectly or destabilize nascent intracellular particles by reducing the availability of ApoE. Interestingly, a similar phenomenon has been reported in GGA1-silenced cells infected with HIV (42). Silencing of GGA2 significantly reduced extracellular infectivity (Fig. 1B), suggesting that this adaptor may link HCV to subsequent secretory compartments. Silencing of GGA3 produced an almost complete loss of both intra- and extracellular infectivity, implicating it as an important mediator of infectious HCV virion assembly. Thus, our observations correlate with the previously reported roles of both the TGN and endosomes in HCV release (43).

DN Rabs with a role in controlling TGN-endosome trafficking (Rab8b, -13, -23, -27, -32, and -33) (Fig. 2) blocked the release of infectious HCV particles, while those involved in internalization/endocytic pathways (e.g., Rab3A, -7, -9, -11, -35, and -37) displayed little effect (Fig. 2). Rab8b, -32, and -33 regulate medial and post-TGN trafficking, while Rab13 controls tight junction biogenesis through TGN-mediated transport (44). Surprisingly, Rab11, known to mediate recycling endosome transport (45), did not influence HCV secretion in our assays despite an earlier siRNA screen identifying a role during secretion of infectious HCV (46). Again, these previous studies differed from ours by assessing the release of chimeric Jc1 (J6/JFH-1), which routinely gives titers up to 1,000-fold higher than those of JFH-1 (24, 47). We propose that different HCV subtypes/genotypes may follow subtly altered pathways during virus egress.

We further demonstrate that VLDL secretion is unaffected by silencing of the DN Rabs and TGN-endosome adaptors that block HCV egress (Fig. 3), while inhibition of ApoE secretion using monensin does not impair core protein release (Fig. 4). This would support a postrelease association between HCV particles and VLDL, rather than the formation of cosecreted, hybrid “lipo-viral particles,” in agreement with the dynamic transfer of HCV infectivity between VLDL and chylomicron compartments within patients (48). This suggests that proteins required for VLDL assembly, including ApoB, ApoE, and microsomal triglyceride transfer protein (MTP), contribute specifically to virion assembly rather than to subsequent virion release from cells although they may also be incorporated into infectious virions (49, 50).

Finally, we observed that the TGN membranes and components redistribute to LD-, ApoE-, and core protein-positive compartments in HCV-infected cells. The TGN redistribution was dependent upon the NS2 protein, but only in the context of a functional replicase, suggesting that NS2 interactions with other nonstructural components mediate the formation of these modified compartments. Consistent with this finding, NS2 has been shown to play a central role during HCV particle assembly and release through its interactions with both structural and nonstructural proteins in close proximity to LDs (7–10). Our data indicate that the redistribution of TGN components to virus assembly sites may be a critical outcome of such interactions.

In conclusion, our results strongly support the idea that the TGN and associated endosomal compartments represent a major route for the trafficking and release of infectious HCV particles from Huh7 cells, which occur independently of VLDL secretion. Moreover, the virus appears to actively modulate these compartments, bringing components of the TGN to the sites of virus assembly through the action of NS2. These data provide further evidence that the mechanism of HCV release requires not only LD-associated endoplasmic reticulum (ER) but also membrane compartments involved in the later secretory pathway. Future studies should focus on elucidating the interactions between viral and host proteins that allow HCV to usurp this pathway.
